# Glucosylceramide synthase inhibition protects against cardiac hypertrophy in chronic kidney disease

**DOI:** 10.1038/s41598-022-13390-z

**Published:** 2022-06-04

**Authors:** Gabriel C. Baccam, Jian Xie, Xin Jin, Hyejung Park, Bing Wang, Hervé Husson, Oxana Ibraghimov-Beskrovnaya, Chou-Long Huang

**Affiliations:** 1 Division of Nephrology, Department of Internal Medicine, University of Iowa Carver College of Medicine, 200 Hawkins Drive, E300 GH, Iowa City, IA 52242-1081 USA; 2 US Early Development, Synthetics Platform, Global CMC Development, Sanofi, Waltham, MA 02451 USA; 3 Genomic Medicine Unit, Sanofi, Framingham, MA 01701 USA; 4 Rare and Neurologic Diseases, Sanofi, Framingham, MA 01701 USA; 5 Dyne Therapeutics, 1560 Trapelo Road, Waltham, MA 20451 USA

**Keywords:** Kidney, Kidney diseases

## Abstract

A significant population of patients with chronic kidney disease (CKD) develops cardiac hypertrophy, which can lead to heart failure and sudden cardiac death. Soluble klotho (sKL), the shed ectodomain of the transmembrane protein klotho, protects the heart against hypertrophic growth. We have shown that sKL protects the heart by regulating the formation and function of lipid rafts by targeting the sialic acid moiety of gangliosides, GM1/GM3. Reduction in circulating sKL contributes to an increased risk of cardiac hypertrophy in mice. sKL replacement therapy has been considered but its use is limited by the inability to mass produce the protein. Therefore, alternative methods to protect the heart are proposed. Glucosylation of ceramide catalyzed by glucosylceramide synthase is the entry step for the formation of gangliosides. Here we show that oral administration of a glucosylceramide synthase inhibitor (GCSi) reduces plasma and heart tissue glycosphingolipids, including gangliosides. Administration of GCSi is protective in two mouse models of cardiac stress-induction, one with isoproterenol overstimulation and the other with 5/6 nephrectomy-induced CKD. Treatment with GCSi does not alter the severity of renal dysfunction and hypertension in CKD. These results provide proof of principle for targeting glucosylceramide synthase to decrease gangliosides as a treatment for cardiac hypertrophy. They also support the hypothesis that sKL protects the heart by targeting gangliosides.

## Introduction

Chronic kidney disease (CKD) is an epidemic affecting ~ 10% of the world population and 15% of the US population, ~ 37 million adults^1^. Patients with CKD can progress to end-stage kidney failure requiring dialysis or kidney transplant^2,3^. Often, CKD patients die from cardiovascular disease before reaching end-stage renal failure. Cardiac hypertrophy leading to cardiac arrhythmia, sudden death, and congestive heart failure are among the most common causes of cardiovascular disease in CKD patients^4^. Cardiac hypertrophy in CKD occurs despite the adequate control of conventional risk factors such as hypertension, anemia, and volume overload, suggesting that CKD-specific factors also exist^3,5,6^. The incidence of cardiac hypertrophy in intermediate stages of CKD is ~ 50–70% and up to 90% in patients with end-stage CKD. Cardiac hypertrophy leading to cardiac arrhythmia, sudden death, and congestive heart failure are among the most common causes of cardiovascular disease in CKD patients^4^. The prevalence of CKD and associated cardiac hypertrophy is on the rise in part due to a growing aging population^7,8^. New treatments to alleviate the burden of the disease are urgently needed.

Klotho is a type-1 membrane protein with a large ectodomain that is predominantly produced in the kidney^9^. The ectodomain is shed into the extracellular fluid as soluble klotho (sKL) and functions as a circulating endocrine and local paracrine hormone^10^. The levels of circulating sKL are reduced in CKD patients and mouse models of CKD^11,12^. We and others have reported that sKL deficiency is an important contributor of CKD-specific factors of cardiac hypertrophy^13^. We have found that cardioprotection by sKL is achieved by regulating the formation and function of lipid rafts^14,15^. Lipid rafts are highly dynamic microdomains of the plasma membrane that are enriched with cholesterol and sphingolipids and can be viewed as a platform for signaling and trafficking^16^. Mechanistically, sKL targets the gangliosides, GM1/GM3, in lipid rafts as its receptor. sKL binds to the α2-3-sialyllactose moiety of clustered gangliosides to regulate the formation of lipid rafts and raft-associated phosphoinoside-3-kinase (PI3K) activity. The transient receptor potential cation channel subfamily C member 6 (TRPC6) is a Ca^2+^-permeable channel present in lipid raft (caveolae) of cardiac myocytes^14,17–19^. Exocytotic insertion of TRPC6 from the sub-membranous to the membranous compartment of the cell requires the activity of PI3K. The promoter of TRPC6 contains a nuclear-factor-of-activated T-cells (*NFAT*)-responsive element is activated by the Ca^2+^-dependent phosphatase, calcineurin^20,21^. Stress-induced pathological Ca^2+^ signaling upregulates TRPC6 and TRPC-mediated Ca^2+^ entry, leading to pathological cardiac remodeling via a vicious feedforward amplification cycle. By downregulating PI3K and TRPC6-mediated pathological Ca^2+^ signaling, sKL protects the heart against stress-induced cardiac hypertrophy^14,15^.

Glycosphingolipids (GSL) are important lipid constituents of the cell membrane^22^. They include cerebrosides (one sugar such as glucose or galactose attached to ceramide), globoside (more than one sugar attached to ceramide), and gangliosides. GSL synthesis begins with glucosylation or galactosylation of ceramide. The formation of glucosylceramide is catalyzed by UDP-Glucose Ceramide Glucosyltransferase (UGCG; also known as glucosylceramide synthase, GCS). Disturbances of GSL metabolism cause many diseases^16,23^. GSL metabolic defects lead to lipid accumulation and lipotoxicity in cells and organs causing diseases such as Gaucher Disease, Fabry’s Disease, and Gangliosidosis^24–26^. Gaucher Disease is caused by the gene mutation of lysosomal acidic glucocerebrosidase (GBA) which normally breaks down membrane glucosylceramide (GlcCer) routed to lysosomes for degradation^27^. Deficiency in GBA leads to cellular GlcCer buildup and toxicity. Cardiac dysfunction can be associated with changes in lipid composition and accumulation, another common occurrence in diseases caused by GBA deficiency. Others have shown that reduction in accumulation leads to improvement in the metabolic pathways associated with cardiac remodeling^28–30^. Inhibitors of glucosylceramide synthase (GCSi) reduces cellular GlcCer content. GCSi has been in use in clinical medicine for > 15 years as a substrate reduction agent as an alternative to enzyme replacement therapy for treating Gaucher Disease.

Our finding that sKL targets gangliosides to exert cardioprotection raises the interesting question to whether GCSi is an effective therapy for treating cardiac hypertrophy. In this study, we demonstrate that GZ667161, a small molecule GCSi, protects the heart against isoproterenol (ISO)-and CKD-induced pathological cardiac dysfunction in mice. The proven safety profile and potential efficacy of GCSi’s in treating human lysosomal storage diseases provides the optimism that GCSi’s, may be a new therapeutic option for cardiac hypertrophy in CKD patients.

## Results

### Glucosylceramide synthase inhibitor reduced TRPC6 current in vitro

**Figure 1:**
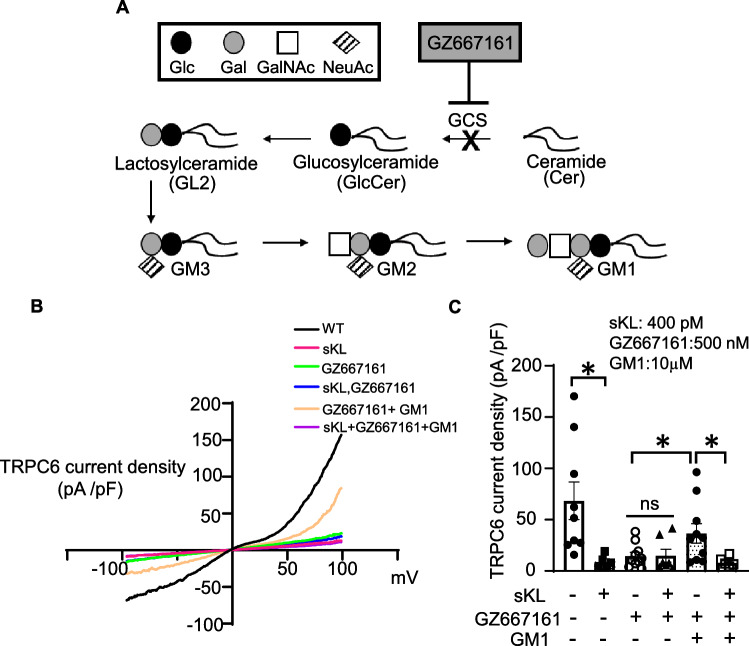
Effect of GZ667161 compound on sKL inhibition of TRPC6 in vitro. (**A**) Simplified diagram of the biosynthesis of gangliosides relevant to this study. Abbreviations: glucose (Glc), galactose (Gal), N-acetyl galactosamine (GalNAc), N-acetylneuraminic acid (NeuAc, sialic acid). (**B**) Representative current–voltage relationship (I-V) curve of TRPC6 current from experiments shown in panel C. (**C**) TPRC6 current in HEK293 cells overexpressing TRPC6 are treated with sKL, GZ667161, and/or GM1 (n = 5–10 per group) *P < 0.05 between indicated groups. ns, not significantly different between indicated groups. Data is expressed as mean ± SEM.

Pathologic cardiac remodeling occurs during times of cardiac stress. TRPC6 is an important player of this process by providing a feedforward amplification cascade. TRPC6 channels are present in lipid rafts (caveolae) of cardiac myocytes;^19^ sKL exerts cardioprotection through targeting sialogangliosides and down regulates raft-associated TRPC6 activity^14,18^. We have previously shown that a GCSi, NB-DGJ (N-(n-Butyl)deoxygalactonojirimycin), inhibits TRPC6 channel similar to sKL by targeting sialogangliosides. GZ667161 is a newly developed GCSi with higher potency (Fig. 1A). Here, we first validated the effect of GZ667161 on TRPC6. As previously described, HEK293 cells expressing recombinant TRPC6 were treated with sKL and/or GZ667161. We observed that TRPC6 current density is significantly reduced when treated with GZ667161 or sKL as compared to no treatment^18^. When cells were co-treated with sKL and GZ667161 there was no additive effect on the TRPC6 current, indicating that they act in the same pathway (Fig. 1B,C). The effect of GZ667161 was due to blocking the synthesis of GM1, as addition of GM1 in GZ667161-treated cells leads to a partial restoration of TRPC6 current. Supporting the notion that sKL inhibits TRPC6 currents by targeting to GM1, we found that TRPC6 currents rescued by GM1 can be inhibited by sKL. These outcomes recapitulate results of our previous studies using NB-DGJ^14^. The potency of GZ667161 is 50-fold higher than for NB-DGJ: the maximal inhibition of TRPC6 occurs at a concentration of 0.5 μM for GZ667161 vs 25 μM for NB-DGJ^14^. Thus, GZ667161 is a good potential candidate for studying cardioprotection in in vivo models of heart failure and CKD.

### GSC inhibitor reduces glycosphingolipids (GSL) in the plasma and heart tissue

**Figure 2:**
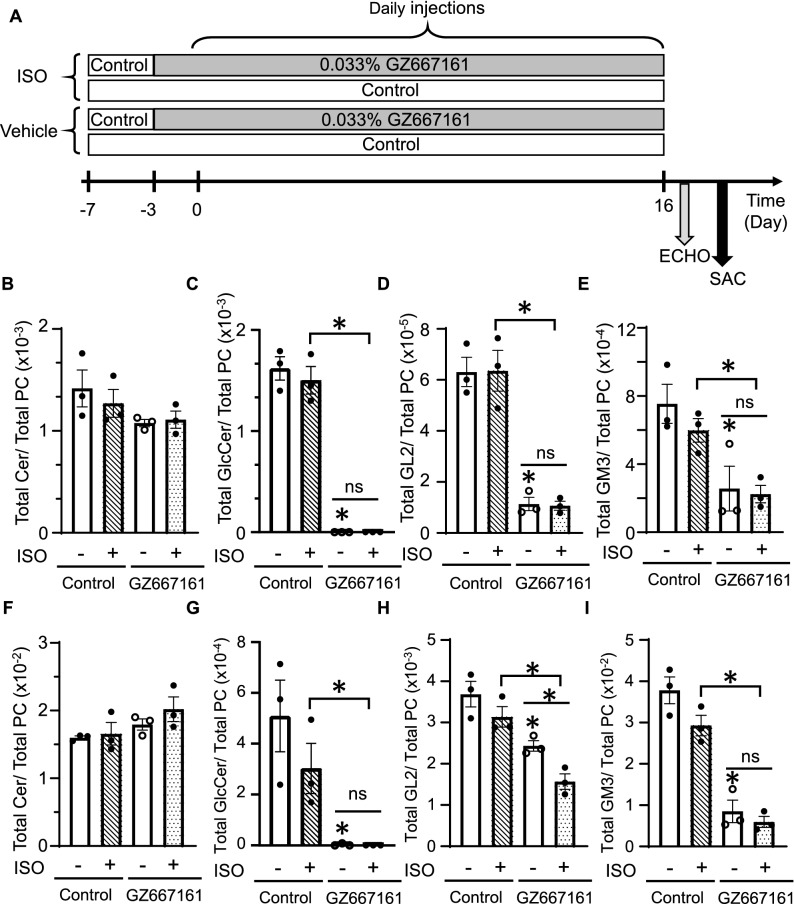
GZ667161 decreases GSL levels in the plasma and heart tissue of +/− ISO treated mice. (**A**) Diagram of experimental design. (**B**) Total ceramide levels normalized to phosphatidylcholine (PC) in plasma. (**C**) Total Glucosylceramide (GlcCer) normalized to control lipid PC in plasma. (**D**) Total GL2 levels normalized to PC. (**E**) Total ganglioside GM3 in plasma normalized to PC. (**F**) Total ceramide levels normalized to PC in heart tissue. (**G**) Total Glucosylceramide (GlcCer) normalized to control lipid PC in the heart tissue of +/− ISO mice. (H) Total GL2 levels normalized to PC. (**I**) Total ganglioside GM3 in the heart normalized to PC. *P < 0.05 between indicated groups or − ISO control. ns not significantly different between indicated groups. Mice were fed control or 0.033% wt/wt GZ667161 diet. (n = 3 per group) Data is expressed as mean ± SEM.

GZ667161 is a potent orally available small molecule inhibitor of GCS^31^. It has recently been used in pre-clinical studies as a substrate reduction therapy effecting the brain. To investigate the effect of GZ667161 on cardioprotection, we studied its effect on Isoproterenol (ISO)-induced cardiomyopathy. ISO overstimulation is a well-accepted stress-induced cardiomyopathy model^32,33^. Age matched mice were fed control diet at day -7, and then separated in two groups at day -3, one group continued on control, and the other one switched to a 0.033% wt/wt GZ667161 diet (Fig. 2A). Thereafter, mice received subcutaneous ISO daily for 16 days. ECHO was performed on mice after the last day of ISO treatment. After 16 days of consecutive treatment of ISO, mice were euthanized, and blood and tissues were collected for analysis.

Plasma levels of total GSL were measured and normalized to total phosphatidylcholine (PC). Ceramide levels between the groups remained unchanged (Fig. 2B) and total glucosylceramide were reduced in mice treated with GZ667161 with or without ISO induction as compared to WT mice on control diet (Fig. 2C). Along with the reduction in glucosylceramide, there was a reduction in total lactosylceramide (GL2) and GM3 levels. (Fig. 2D,E). In heart tissue of WT mice treated + /- ISO and given a GZ667161 diet, ceramide levels remained unchanged, while a reduction of total glucosylceramide levels was present and met with a reduction of both total GL2 and GM3 (Fig. 2F-I). These results confirm that treatment with GZ667161 leads to reduction of glycosphingolipids (GSL) in vivo by blocking the synthesis of glucosylceramide from ceramide.

### GZ667161 protects against ISO-induced cardiac remodeling

**Figure 3:**
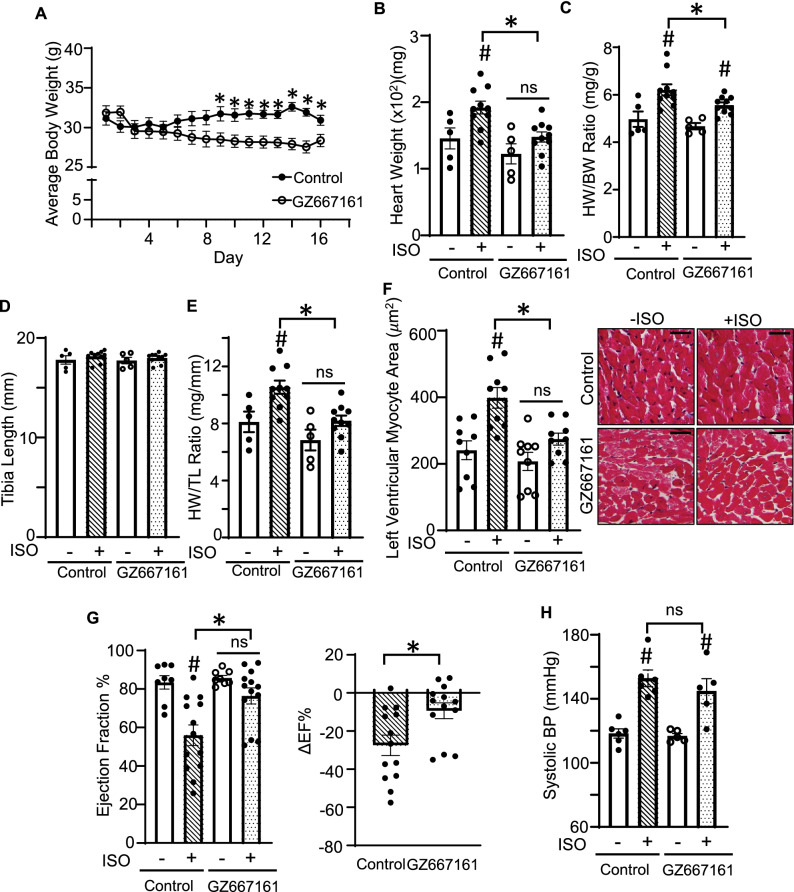
GZ667161 compound ameliorates ISO-induced cardiac remodeling. (**A**) Time course of average body weight of ISO treated mice (n = 10 per group) fed with control or GZ667161 diet. (**B, C**) Heart weight (**B**) and HW/BW ratio (**C**) in control or GZ667161 diet with or without ISO. − ISO (n = 5 per group). + ISO (n = 10 per group). (**D**, **E**) Tibia length (**D**) and HW/TL ratio (**E**) in control or GZ667161 diet with or without ISO. (**F**) Left ventricular myocyte area. (**G**) Ejection fraction and difference in ejection fraction and change in ejection fraction percentage as compared to average control for respective groups. − ISO (n = 8 per group), + ISO (n = 13 per group). (**H**) Systolic blood pressure of the mice. Data is expressed as mean ± SEM. #*P* < 0.05 − ISO versus + ISO. *P < 0.05 between indicated groups. ns, not significantly different between indicated groups.

Having shown that GZ667161 significantly reduces GSL levels in the heart and in plasma of treated mice, we next examined its effect on heart weight/body weight (HW/BW) ratios, an index to measure cardiac hypertrophy. Mice on 0.033% GZ667161 diet showed a significant decrease in body weight (BW) compared to mice on control diet (Fig. 3A). As expected, ISO treatment significantly increased heart weight and HW/BW ratios in mice on control diet indicating hypertrophy (Fig. 3B,C). ISO treatment also induced cardiac hypertrophy in mice fed on GZ667161 diet (“ + ISO” vs “-ISO”), though the degree of ISO-induced hypertrophy was moderately reduced in GZ667161-treated vs control diet (“GZ667161 + ISO” vs “control + ISO”). Body weight loss in GZ667161-treated mice may have confounded the analysis of HW/BW ratios. GZ667161 treatment did not affect tibia length (TL) (Fig. 3D). Thus, we normalized HW to tibia length (TL) (Fig. 3E)^34^. In mice fed on control diet, ISO overstimulation increased HW/TL, confirming cardiac hypertrophy. There were no significant differences in HW/TL between ISO- vs vehicle-treated mice fed with GZ667161, indicating that GZ667161 diet ameliorated ISO-induced cardiac hypertrophy. Furthermore, left ventricular myocyte area was increased by ISO and ISO-induced increases were reduced by GZ667161 treatment (Fig. 3F). ECHO revealed that ISO treatment caused functional decline (decreases in ejection fraction) in control mice but not in GZ667161-treated mice (Fig. 3G). Therefore, along with the effect on cardiac mass, GZ667161 also prevented ISO-induced cardiac function decline. The effect of GZ667161 is not mediated through changes in blood pressured (Fig. 3H).

**Figure 4:**
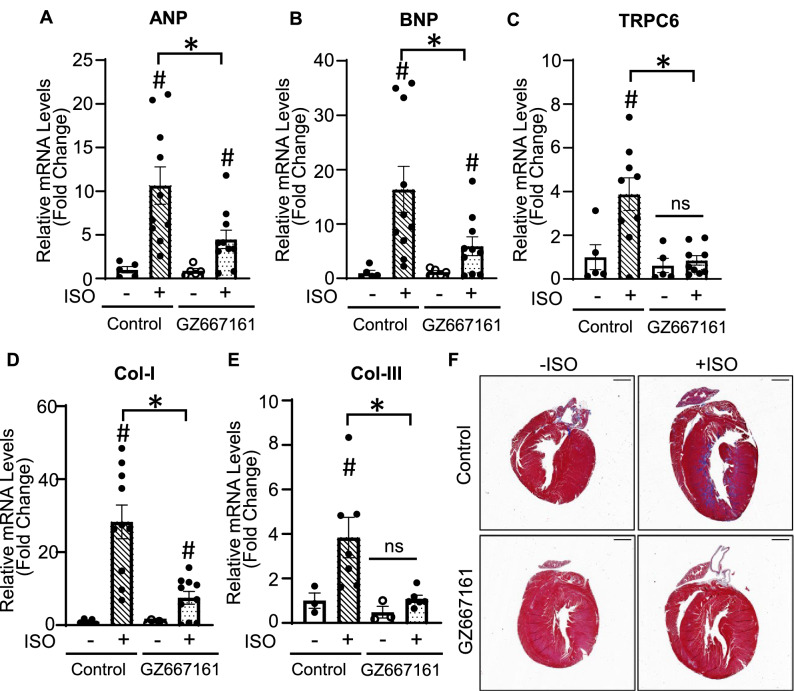
GZ667161 compound reduces expression of cardiac remodeling genes in the heart. (**A-E**) RNA from heart tissue was isolated and RT-qPCR was performed to examine the expression of cardiac remodeling genes in the heart in mice treated with or without ISO after 16 days of treatment. (**A**) ANP, (**B**) BNP, (**C**) TRPC6, (**D**) Col-I. (**E**) Col-III. (**F**) Representative trichrome staining for collagen fibers on ISO treated or untreated mice. Scale bar is 1 mm. − ISO (n = 5 per group). + ISO (n = 9–10 per group) #*P* < 0.05 − ISO vs + ISO. *P < 0.05 between indicated groups. ns, not significantly different between indicated groups. Data is expressed as mean ± SEM.

During times of cardiac stress, cardiac remodeling and cardiac growth occurs through re-expression of fetal heart genes, such as atrial natriuretic peptide (ANP) and brain natriuretic peptide (BNP), which are generally turned off at birth. The expression of TRPC6 in the normal heart is very low. However, it is upregulated during cardiac stress by the pathologically activated Ca^2+^-calcineurin-NFAT signaling cascade. Fibrosis and increased expression of collagen-I (Col-I) and collagen-III (Col-III) are consequences and hallmarks of pathological cardiac hypertrophy. We examined the effects of GZ667161 on genes associated with cardiac remodeling including cardiac fetal genes (ANP and BNP), TRPC6, and Col-I, -III by using quantitative RT-qPCR. Results revealed that ISO overstimulation in mice fed with control diet induced a significant increase in expression of ANP, BNP, TRPC6, and Col-I, -III (Fig. 4A–E). Feeding mice GZ667161 diet, blunted the ISO-induced increases in gene expression. To test the extent of fibrosis in the hearts of mice we performed a trichrome staining to measure for collagen, shown in blue. We found that GZ667161 reduced fibrosis in the hearts of mice as compared to + ISO control mice (Fig. 4F).

### GZ667161 exerts cardioprotection on CKD mice and reduces CKD induced heart failure

**Figure 5:**
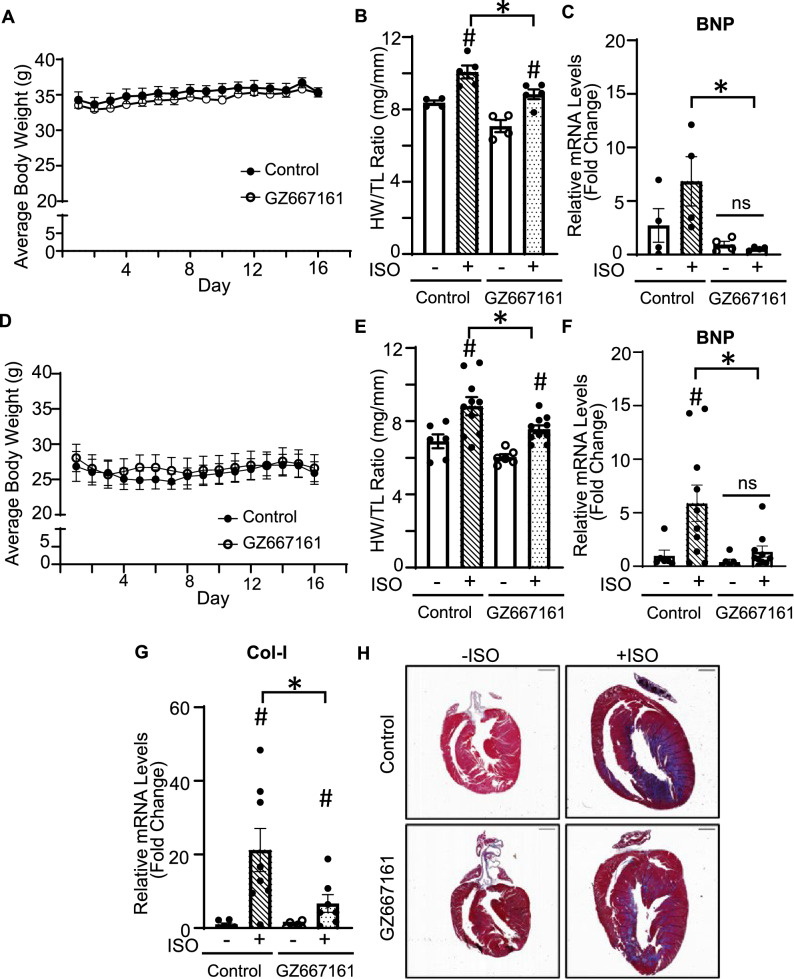
Gavage feeding GZ667161 at a dose of 20 mg/kg does not cause BW loss yet protects the heart. (**A**–**D**) Mice were treated with or without ISO to induce cardiac stress to investigate the role of GZ667161. (**A**) BW time course for treatment with 20 mg/kg GZ66761 through gavage feeding. (**B**) HW/TL ratio. (**C**) Reduction of cardiac remodeling gene BNP. (n = 4–5 per group) (**D**–**G**) Dietary feeding GZ667161 (0.11% wt/wt) provides cardioprotection in ISO-induced model of cardiac stress. Mice were treated with or without ISO to induce cardiac stress to investigate the role of GZ667161. (**D**) Body weight time course for treatment with 0.011% GZ667161 diet. (**E**) HW/TL ratio of mice fed with 0.011% GZ667161 diet. (**F**) BNP. (**G**) Col-I. (**H**) Trichrome staining for collagen fibers on +/− ISO treated mice. Scale bar is 1 mm. + ISO (n = 10 per group). − ISO (n = 6 per group). #*P* < 0.05 − ISO vs + ISO. *P < 0.05 between indicated groups. ns, not significantly different between indicated groups. Data is expressed as mean ± SEM.

sKL protects CKD-associated cardiomyopathy by targeting GSL gangliosides, therefore providing a basis to examine the effect of GZ667161 on CKD-induced stress and dysfunction using a 5/6 nephrectomy mouse model. In pilot experiments we found CKD mice do not tolerate the same dose of GZ667161 as used for ISO studies (0.033% wt/wt) (not shown). Uremic environment may contribute to the reduced tolerance to GZ66716. Hence, we performed experiments testing the effect of reduced dose in our ISO overstimulation model. We first used gavage feeding to eliminate potential dosage variation from variable food intake. Gavage feeding at 20 mg/kg of GZ667161 (equivalent to 0.011% wt/wt) did not cause weight loss yet conferred cardioprotection (Fig. 5A–C). We found that gavage feeding 20 mg/kg significantly decreased HW/TL and expression of BNP compared control diet in ISO overstimulation model (Fig. 5B,C). We next examined the effects of dietary feeding of the reduced dose GZ667161 (0.011% wt/wt) and confirmed that this reduced dose prevented body weight loss as compared to the higher dose (0.033% wt/wt) (Fig. 5D) while conferred moderate protection against ISO-induced increases in heart mass index, BNP gene expression, Col-I expression, and fibrosis (Fig. 5E–H). Having confirmed that 0.011% GZ667161 diet is effective in ISO-induced model, we used the reduced dose in CKD studies.

**Figure 6:**
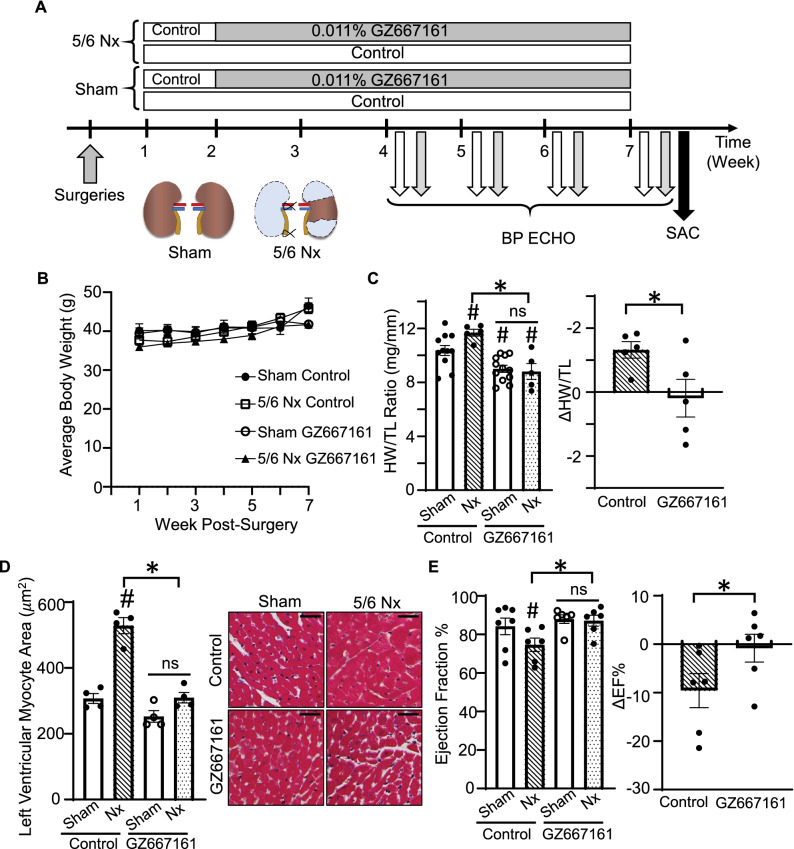
GZ667161 compound exerts cardioprotection on 5/6 Nephrectomy (Nx) Mice. (**A**–**D**) Mice underwent 5/6 nephrectomy or sham surgery operation. Mice were monitored for 7 weeks. (**A**) Diagram of experimental design for 5/6 Nx mice. (**B**) Average body weight time course post-operation (n = 7,6 sham control, CKD control respectively) (n = 7,5 sham GZ667161, 5/6 Nx GZ667161, respectively). (**C**) HW/TL ratio of mice collected after euthanasia at 7 weeks and the difference in HW/TL between the average HW/TL of 5/6 Nx and sham mice feed with control diet. (n = 11,5 sham control, CKD control respectively) (n = 12,5 sham GZ667161, 5/6 Nx GZ667161, respectively). (**D**) Left ventricular myocyte area. (**E**) Ejection Fraction of sham and control mice measured through ECHO and the difference in EF% in mice compared to control diet fed mice in the respective groups. (n = 7,6 sham control, CKD control respectively) (n = 6,6 sham GZ667161, 5/6 Nx GZ667161, respectively) #*P* < 0.05 vs. sham control, *P < 0.05 between indicated groups. ns, not significantly different between indicated groups. Data is expressed as mean ± SEM. Note: CKD studies used 0.011% GZ667161 diet.

**Figure 7:**
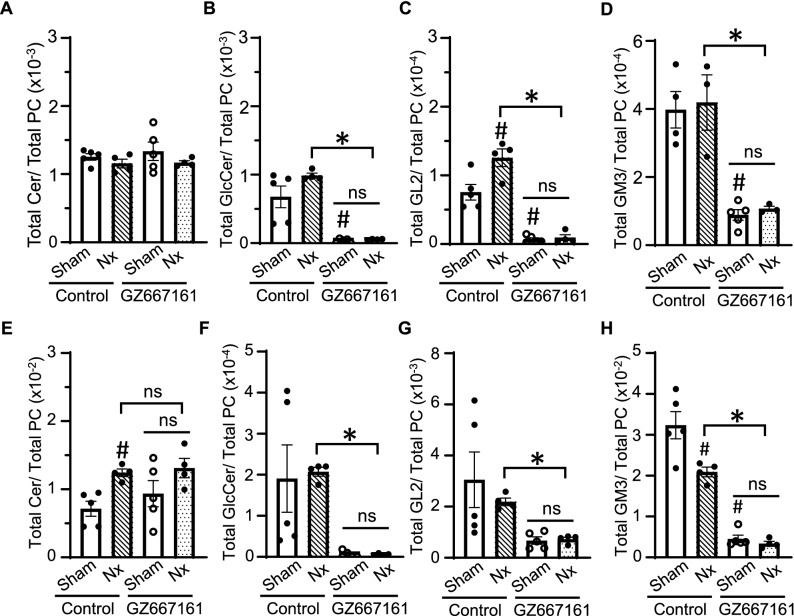
Reduced dose GZ667161 decreases GSL levels in the heart and plasma of CKD and sham mice. (**A**–**D**) Total ceramide, glucosylceramide, GL2, and ganglioside GM3 levels normalized to PC in the plasma of sham mice on control diet. (**E**–**H**) Total ceramide, glucosylceramide, GL2, and ganglioside GM3 levels normalized to PC in heart tissue of sham mice on control diet. Sham (n = 5 per group); 5/6 Nx (n = 4 per group). #*P* < 0.05 vs. sham control of respective group. *P < 0.05 between indicated groups or sham control. ns not significantly different between indicated groups. Data is expressed as mean ± SEM.

Age-matched mice received sham or 5/6 nephrectomy procedure. Mice were switched from regular rodent chow to control diet 1-week post- surgery and then one group was switched to GZ667161 or kept on control diet 2-weeks post-surgery. Mice received weekly blood pressure and ECHO measurement between 4 and 7 weeks. At the end of 7 weeks post-surgery mice were euthanized for blood and tissue collection (Fig. 6A). At the reduced dose of GZ667161, we observed no significant changes or differences in body weight after recovery from CKD surgery between the 4 groups (Fig. 6B). As expected, relative to mice that received sham operation, CKD mice fed with control diet developed cardiac hypertrophy as reflected by increases in HW/TL and left ventricular myocyte area (Fig. 6C,D). CKD-induced cardiac dysfunction was blunted by feeding on GZ667161. Functional studies by ECHO revealed that CKD caused impairment in cardiac function in mice with control diet as evident by a decline in ejection fraction (Fig. 6E). CKD-induced cardiac functional decline was ameliorated by feeding CKD mice GZ667161 diet. At the reduced dose, GZ667161 also effectively inhibited GCS and reduced downstream GSL levels in in the plasma and heart tissue of mice (Fig. 7A–H).

**Figure 8:**
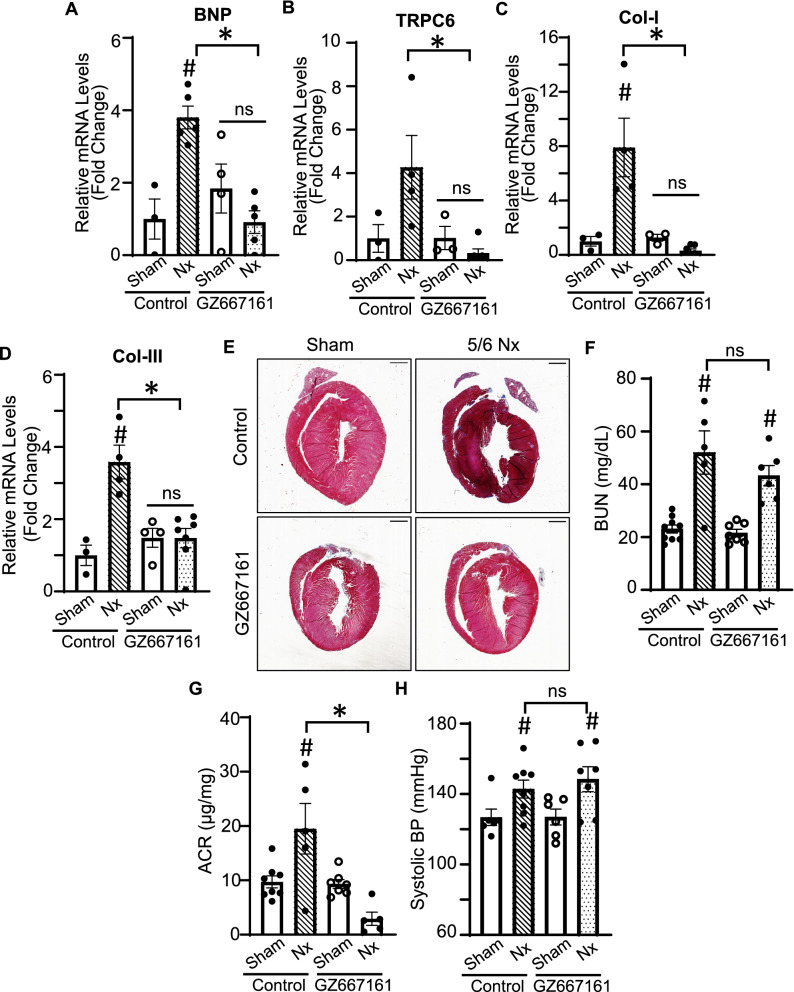
GZ667161 ameliorates expression of cardiac remodeling genes in CKD mice. (**A**–**D**) RNA from heart tissue was isolated and RT-qPCR was performed to examine the expression of cardiac remodeling genes in the heart in mice with 7 weeks post-5/6 Nx operation. (**A**) BNP. (**B**) TRPC6. (**C**) Col-I. (**D**) Col-III. (**E**) Representative trichrome staining of CKD hearts. Scale bar is 1 mm. Sham (n = 3–4 per group). CKD (n = 3–4 per group). GZ667161 compound does not impact or change physiological conditions of CKD. (**F**) Blood urea nitrogen levels. (n = 9,5 sham control, CKD control respectively) (n = 7,6 sham GZ667161, 5/6 Nx GZ667161, respectively). (**G**) Albumin creatinine ratio (ACR). (**H**) Systolic BP, 4 weeks post-operation shown. Sham (n = 6), 5/6 Nx (n = 7–8). #*P* < 0.05 Sham vs. 5/6 Nx in respective groups. #*P* < 0.05 vs. sham control of respective group. *P < 0.05 between indicated groups. ns, not significant between indicated groups. Data is expressed as mean ± SEM.

The effects on cardiac function and heart mass were further corroborated by studies on gene expression. As shown, CKD induced upregulation of BNP in mouse hearts fed on control diet (Fig. 8A). The increases in gene expression were blunted by feeding on GZ667161. CKD also increased TRPC6 expression cardiac fibrosis as evident by increased Col-I and -III expression and trichrome staining, which were blunted by GZ667161 diet (Fig. 8B–E). Overall, our results show that GZ667161 protects the heart against CKD-induced cardiac dysfunction. CKD-induced hypertension and biochemical disturbances may contribute to cardiac hypertrophy. To know whether the cardioprotective effect of GZ667161 may be related to these factors, we compared these parameters in CKD mice fed on control and GZ667161. As shown, clearance function reflected by degrees of azotemia (increased BUN level) was not different between CKD mice on control vs GZ667161 diets (Fig. 8F). We also found that water intake and urine output for CKD mice were increased compared to the sham controls for the respective groups but were not significantly different between the CKD groups (not shown). GZ667161 treatment did ameliorate CKD-induced increased urinary albumin excretion (Fig. 8G), but had no effect on CKD-associated increases in systolic blood pressure (Fig. 8H). CKD by 5/6 nephrectomy did not cause TRPC6 upregulation in the whole kidney tissue (Supplementary Fig. 1A). TRPC6 upregulation is due to stress causes abnormal intracellular Ca^2+^ increases^13–15,18^. As TRPC6 has NFAT-response element in the promoter, the initial abnormal intracellular Ca^2+^ increases trigger a feedforward TRPC6 amplification and upregulation. We have found that lipid raft-associated PI3K is important for TRPC6 insertion to lipid rafts. GZ667161 ameliorates TRPC6 upregulation presumably by inhibiting lipid raft formation. As shown, in the absence of stress-induced upregulation, GZ667161 treatment had no effect on basal TRPC6 level in the kidney (Supplementary Fig. 1A). GZ667161 treatment prevented downregulation of klotho in CKD and ameliorated renal fibrosis in 5/6-nephrectomized remnant kidney tissues as shown by Col-I, Col-III, and trichrome staining (Supplementary Fig. 1B–F).

## Discussion

Glycosphingolipids (GSL) including cerebrosides, globoside, and gangliosides are important lipid constituents of the cell membrane. Genetic mutation of enzymes involved in GSL metabolism cause lysosomal storage diseases^35,36^. These diseases, such as Gaucher, Fabry’s, Gangliosidosis, are results of cell/organ-toxicity from lipid overload. Enzyme replacement therapy is the mainstay therapy for these diseases. However, administered enzymes may not reach the organs to which they should target. Alternatively, the employment of substrate reduction therapy aims to reduce lipid overload in target organs by the use of small molecule drugs. Glucosylceramide synthase is the enzyme for the entry pathway for GSL synthesis. Many inhibitors of GCS (GCSi) including NB-DNJ (Miglustat), NB-DGJ (Lucerastat), Eliglustat and Venglustat, have been used or are under clinical trial for treating these diseases, supporting their safety profile and efficacy in clinical applications^37–40^.

Patients with CKD have an increased risk of developing cardiac hypertrophy despite adequate control of conventional risk factors such as hypertension, volume overload, and anemia. Previously we have shown in a mouse model of CKD that sKL deficiency is an important contributing factor^6^. TRPC6-mediated pathological calcium signaling plays an important role in feed-forward amplification of pathological cardiac remodeling. PI3K is essential for exocytotic insertion of TRPC6 channels^14,15,18^. Mechanistically, sKL targets to gangliosides in membrane lipid rafts to affect raft-associated PI3K and TRPC6 exocytosis to exert cardioprotection. Replacement by recombinant sKL is limited by the challenges of mass protein production. Thus, we tested whether pharmacological manipulation of membrane ganglioside content may be helpful for treatment of stress-induced cardiac dysfunction. Using two models of cardiomyopathy, isoproterenol overstimulation and a 5/6 nephrectomy CKD model, we showed that a newly developed GCSi, GZ667161, confers cardioprotection in both models. Compared to other GCSi such as, NB-DGJ (Lucerastat) which is currently in phase III clinical trial, GZ667161 inhibits sKL-mediated regulation of TRPC6 with ~ 50-fold higher potency. With respect to the effects on the kidney, GZ667161 treatment reduces fibrosis in the remnant tissue of 5/6-nephrectomized kidney. The protection against renal fibrosis may be related to amelioration of the downregulation of klotho in CKD. The detailed mechanism of how GZ667161 reduces klotho downregulation is unknown and remains to be investigated. GZ667161 treatment however does not affect CKD-induced urea clearance and CKD-associated hypertension. The possibility that GZ667161 affects other factors that impact cardioprotection, such as circulating FGF23 levels cannot be eliminated. Overall, the findings in the ISO-induced model supports the notion that GZ667161 can exert cardioprotective actions independently of renal protection.

Our study is the first to report the use of GCSi as an effective therapy without the presence of accumulation or abnormal levels of GSL in disease. In our study, GSL contents in the plasma and heart tissue are not increased in CKD or ISO-treated vs sham or non-treated mice. GCSi protects the heart by modulating GSL-dependent membrane signaling rather than correcting lipid overload. With regard to cardioprotection by GCSi, a previous study by Mishra et al. reported that high-fat and high-cholesterol diets cause GSL overload in the hearts of *ApoE*-deleted mice and these mice develop cardiomyopathy. Treatment with D-PDMP (D-threo-1-phenyl-2-decanoylamino-3-morpholino-1-propanol) inhibits glucosylceramide synthase and lactosylceramide synthase, decreasing GSL overload and improving left ventricular mass and function in these mice^41^. Our study and the report by Mishra et al. strongly support the notion that decreasing cardiac gangliosides using GCSi confers protection against pathological cardiac hypertrophy. Our study demonstrates the efficacy of GCSi on stress-induced cardiomyopathy not originating from cardiac lipid overload and lipotoxicity. The proven safety profile of GCSi and higher potency raises the enthusiasm that GZ667161 or related GCSi may be a new therapy for causes of cardiac hypertrophy including cardiac hypertrophy in CKD patients.

A recent study reported that mice with homozygous deletion of *Ugcg* encoding GCS in myocytes using α-*MHC*-Cre (*Ugcg*^*f/f*^; α-*MHC*-Cre) developed dilated cardiomyopathy and premature death^23^. Of note, lipid rafts are ubiquitously present in eukaryotic cells. They are membrane platforms for growth factor receptors and associated signaling complexes, and for membrane trafficking^42^. The specific lipid raft type present in cardiomyocytes, caveolae, also play an important role in the ultrastructure of myocytes. α-*MHC* expression begins very early in embryonic development in mice, at around embryonic day 8.5 and increases at neonatal day 2^43–45^. It is not surprising that deletion of GSL and lipid rafts early in development has detrimental effects in mice.

Oral administration of GCSi lowers GSL systemically and in many tissues as evident by the reduction in the plasma levels. While we show that GSL contents are reduced in the heart, we cannot exclude that cardioprotection occurs indirectly through other organs. Our results suggest that it is not through renal protection or improvement of CKD-induced hypertension. GZ667161 crosses the blood brain barrier and reduces GSL in CNS^31^. Thus, the possibility that an effect on CNS and neural control of heart function yet exists.

Lipid rafts are GSL- and cholesterol-enriched microdomains in which the lipid constituents are in dynamic equilibrium with those in the bulk membrane^42^. Formation of lipid rafts is governed by physicochemical properties of lipids and stabilized by local lipid–protein and protein–protein interactions^46^. The density of lipid rafts varies among cell types ranging from fractions of a percentage to a few percentages of total cell membrane^47^. As membrane platforms for signaling complexes and trafficking, lipid rafts are essential for cell and organism function. They are also targets for modulating cellular signaling cascades. The effect of sKL on raft-associated gangliosides does not result in an all-or-nothing function of lipid rafts in cells. Rather, sKL regulates the size and/or number of lipid rafts present. The abundance of gangliosides within the lipid raft increases when size of the lipid raft grows. This increases the local concentration of gangliosides and enhances the binding of sKL to gangliosides within rafts. sKL binding to gangliosides leads to dissociation and downsizing of lipid rafts. Thus, through regulating lipid raft formation sKL modulates raft-associated growth factor signaling. The physiological effect of sKL on cells and organs depends on lipid raft composition and association to signaling complexes of each cell type. The same principle applies to the pharmacological manipulation of lipid rafts by GCSi. As demonstrated in our pre-clinical studies, a therapeutic dose of GZ667161 is effective in cardioprotection and possesses a good safety profile.

Aside from the cardiac stress-induced model, the baseline heart mass (i.e., -ISO or sham) in GZ667161-treated mice (while not statistically significant) tends to be lower than control fed mice (see Figs. 5B,E and 6C). Mouse organs continue to grow several months after birth. Through its effect on raft-associated growth factor signaling, GZ667161 may slow postnatal cardiac growth. We found that food intake is not different between feeding control vs GZ667161 diet on 0.011% GZ667161 but is different in 0.033% GZ667161 (not shown). Weight loss in mice that received a higher dose of GZ667161 may be due to this decrease in food intake but could also be related to similar effects on growth factor signaling. Whether these combined effects are applicable to adult human hearts is unknown. Nonetheless, it raises interesting questions to whether GCSi may be more broadly applicable in controlling non-stress-induced cardiac growth and hypertrophy.

In summary, this current study provides pre-clinical data that pharmacological inhibition of GCS by GZ667161 ameliorates ISO-induced and CKD induced cardiac dysfunction. This occurs in the setting of no overt lipotoxicity from GSL overload. The data presented provides robust in vivo evidence supporting a potential novel clinical therapeutic for cardiac dysfunction in CKD patients. The data are consistent with our working hypothesis that targeting gangliosides, as believed by sKL, is an effective therapeutic approach for cardioprotection.

## Methods

### Whole-cell recording

For whole-cell recording of recombinant TRPC6 channels overexpressed in HEK293 cells, the pipette and bath solution contained (in mM) 120 Cs-aspartate (Cs-Asp), 10 CsCl, 1 MgCl_2_, 2 MgATP, 5 EGTA, 1.5 CaCl_2_ (free [Ca^2+^] = 70 nM) and 10 CsHEPES (pH 7.2) and 140 NaCl, 5 KCl, 0.5 EGTA and 10 NaHEPES (pH 7.4), respectively. As previously described, whole-cell currents were recorded under voltage-clamp using an Axopatch 200B patch-clamp amplifier (Axon instruments Inc., Foster City, CA, USA)^18,48^. Voltage step protocol includes holding at 0 mV, and 200 ms steps from -100 mV to + 100 mV in + 2 mV increments. The pipette resistance was ~ 2–3 MΩ when filled with the pipette solution. Whole-cell access resistance was < 10 MΩ. Low-pass currents were filtered at 2 kHz and sampled every 0.1 ms. Data acquisition was performed using pClamp9.2 program (Axon Instrument, Inc. Foster City, CA, USA) and analysis using Prism (V8.0) software (GraphPad Software, San Diego, CA, USA)^18,48^.

### Isoproterenol (ISO) induction model of cardiac hypertrophy

Age-matched male 129/SvEv mice (Jackson Laboratory, Bar Harbor, ME, USA) of ~ 12 weeks old were used for ISO model experimentation. Mice were started on PicoLab® Rodent Diet 5053 (control diet) for 1 week before daily injections, and the drug groups were switched on 0.033% wt/wt GZ667161 diet (compound weight/food weight). Induction of cardiac hypertrophy was executed 3 days after diet switch by using ISO (3 mg/kg per day, diluted in normal saline) subcutaneous injection for 16 consecutive days. Blood pressure was taken before and after ISO injection by tail-cuff sphygmomanometer as previously described^18^. Echocardiogram (ECHO) to measure cardiac function was performed on day 17, then mice were weighed and euthanized for tissue collection using Isoflurane and cervical dislocation as approved by University of Iowa Institutional for Animal Care and Use Committee (IACUC).

### CKD model generated by 5/6 nephrectomy

CD-1 male mice (Charles River, Wilmington, MA, USA) ~ 10 weeks old were used for CKD model experiments. 5/6 nephrectomy surgery was performed as described before^6,49^. Mice were anesthetized with 5% isoflurane inhalation (AKORN Inc, Lake Forest IL, USA), and with continued 2% isoflurane inhalation flow throughout the surgery. The midabdominal region was cleaned with alcohol and sanitized with betadine solution. A ~ 2 cm incision was made to open up the abdomen. Using cotton tipped applicators, the left kidney was exposed and isolated. The upper and lower poles were cauterized, leaving approximately 1/3 of the left kidney. Then the right kidney was exposed. The right ureter and renal artery were tied off, and the right kidney was excised and removed. The surgical incision was sutured, and tissue glue was used to secure the closure. The mice were given 5 mg/kg of Carprofen (Zoetis Inc, Kalamazoo, MI, USA) for post-operative pain. Sham mice were generated by making and closing the incision without cauterization or removal of the kidneys. The mice were put on control diet 1-week post-operation. Week 2 post-operation one group of mice were left on control diet and another group was switched to 0.11% wt/wt GZ667161 diet. Blood pressure was measured, and urine was collected at Week 5 post-operation. ECHO was performed at Week 6 post-operation and mice were dissected for tissue collection.

### Measurement of kidney function of CKD mice

Sham and CKD mice were placed unrestrained in metabolic cages for urine collection. 24-h and 48-h urine volume and water intake were measured by weight change of urine collection tubes or water bottles (g) converted to milliliters (mL). Urine was analyzed to examine albumin excretion, measured by albumin-creatinine ration (ACR), using QuantiChrom™ BCG Albumin and Creatinine Assay Kits (BioAssay Systems, Hayward, CA, USA). Blood was collected through retro-orbital bleeding with capillary tubes. Hematocrit was measured using HemataStat II Hematocrit Analyzer (EKF Diagnostics, San Antonio, TX, USA). Plasma was extracted after centrifugation at 5,000 rpm for 5 min. BUN was measured using Pointe BUN MedTest (B7552-150, Canton, MI, USA).

### General experimental procedure on mice

Systolic blood pressure and heart rate were measured non-invasively using tail cuff sphygmomanometer as previously described (Visitech BP-2000 Series IIBlood Pressure Analysis System)^18^. Echocardiogram imaging was performed using Acuson Sequoia model 256 clinical echocardiograph (Siemens, Malvern, PA,USA) fitted with an 8-MHz sector-scanning probe as described without the use of ketamine^50^. End-diastolic volume and stroke volume were measured in order to obtain ejection fraction percentage. Tissues (heart, tibia, and plasma) were collected at the end point. Body weight and heart weight were measured. Portions of heart tissue were collected for RNA isolation (saved in RNAlater), and lipid analysis (snap frozen and stored in -80 °C). The rest of the heart was stored in formalin for histological purposes. Tibia bones were digested with 1% SDS and 0.2 mg/kg proteinase K at 55ׄ°C overnight. Muscle and tissue were gently removed, and tibia was air-dried at room temperature. Tibia length was then measured using a digital caliper. All animal procedures were approved by IACUC and carried out in compliance with ARRIVE guidelines.

### GSL analysis

Tissues utilized in this analysis were collected from ISO treated mice fed control or 0.033% GZ667161 Diet. Sphingolipid quantitative analysis was executed by liquid chromatography and high- resolution or tandem mass spectrometry (LC/HR-MS or LC/MS/MS) as previously described^51^. GlcCer and PC extractions, tissue homogenization protocol, analyses, and modifications for GM3 analysis were also conducted as previously detailed. PC was coextracted with sphingolipids for the purpose of normalization^51,52^. In order to extract GM3, approximately 10 µL of heart tissue homogenate (from approximately 100 mg heart tissue per sample) or 10 µL of serum plasma with a solvent comprised of equal volumes of acetonitrile and methanol. GM3 standard was purchased from Matreya, LLC. GM3 was separated with an isocratic elution using a mobile phase acetonitrile: methanol: water (61.5:33.23:5 vol/vol) in 10 mM of ammonium acetate, pH 6.8 for 2 min using a Waters Acquity UPLC and Waters Atlantis HILIC Silica column (3 mm, 2.1 × 100 mm) at room temperature. The flow rate was 0.4 mL/min. The eluent was analyzed by a Q Exactive mass spectrometer (ThermoFisher Scientific) that is equipped with a Heated ElectroSpray Ionization source. Data were gathered in full MS negative mode using the following parameters: scan range of 1100–1700 m/z, resolution 70,000, AGC target of 3e6, and max IT of 256 ns. Quantification of distinct isoforms with various acyl-chain lengths was exchanged by MS1 extraction of ion chromatograms^52^.

### RNA isolation and RT-qPCR

RNA was extracted from heart tissue using Trizol (Invitrogen) Reagent. RNA (10 mg) samples were then treated with TURBO DNase (Cat #AM1907, Invitrogen) prior to cDNA synthesis. cDNA for each sample was synthesized using 1 mg of RNA with iScript cDNA Kit (BioRad) performed in a BioRad Thermocycler. cDNA was diluted and RT-qPCR was performed in a 96 well-plate with SYBR Green PCR mix. The following primers were used: 18S: F-5ʹ AGGGGAGAGCGGGTAAGAGA-3ʹ, R-5ʹ GGACA GGACTAGGCGGAACA 3ʹ, ANP: F-5ʹ GCCATATTGGAGCAAATCCT 3ʹ, R-5ʹ GCAGGTTCTTGAAA TCCATCA 3ʹ, BNP: F-5ʹ AGGTGCTGTCCCAGATGATTCTGT3ʹ, R-5ʹ CTTGTGCCCAAAGC AGCTTGAGAT 3ʹ, Col-I: F 5ʹ GAGCGGAGAGTACTGGATCG 3ʹ, R-5ʹ GTTCGGGCTGATGTAC CAGT 3ʹ, Col-III: F 5ʹ CCTGGCTCAAATGGCTCAC 3ʹ, R- CAGGACTGCCGT TATTCCCG 3ʹ, TRPC6**:** F-5ʹ CGCTGCCACCGTATGG 3ʹ, R- 5ʹ CCGCCGGTGAGTCAGT 3ʹ, Klotho: F-5ʹ TTCAAACCCGGAAGTC TTTG 3ʹ, R- 5ʹ CCAGGCAGACGTTCACATTA 3ʹ. Primers supplied by Integrated DNA Technologies, Coralville, IA, USA.

### Histology

Hearts were stored and fixed in formalin at 4 °C and then paraffin embedded. Paraffin blocks were sectioned into 5 µm sections using Leica paraffin microtome in the Center of Microscopy at the University of Iowa. Sections were mounted onto Fischer brand frosted plus slides. Slides were then deparaffinized with rinses of xylene and stained with Masson’s Trichrome staining for collagen (blue), muscle fibers (red), and nuclei (black/blue). Coverslips were then mounted on stained slides with Permount Mounting Medium. Slides were then scanned in slide scanner, PrimeHisto XE (Pacific Image Electronics, New Taipei City, Taiwan). For further magnification of cardiomyocytes, slides were viewed under compound light microscopy at 20X. Myocyte size and number were quantified using ImageJ (NIH, Bethesda, MD, USA).

### Statistical analysis

Statistical comparisons were made using student t-tests between control and experimental groups using GraphPad Prism. Each experiment was repeated at least once at different times with similar results. Data are presented as means ± SEM.

### Ethics approval and consent to participate

The experimental protocols were approved and reviewed by University of Iowa Institutional for Animal Care and Use Committee (IACUC) and performed in conformity with ARRIVE guidelines.

### Ethics declarations

All animal studies were performed in accordance with the approved guidelines and protocols by University of Iowa Institutional for Animal Care and Use Committee (IACUC). Protocol #0,041,990.

## Supplementary Information

Supplementary Figure 1.

## Data Availability

The datasets supporting the conclusions are included within this article or are available from the corresponding author upon request.
