# Supplementary material for: Glucosylceramide synthase inhibition protects against cardiac hypertrophy in chronic kidney disease
Source: Sci Rep. 2022 Jun 4;12:9340. doi: 10.1038/s41598-022-13390-z (PMC9167280; doi:10.1038/s41598-022-13390-z)
Supplement: Supplementary file 1 — Supplementary Figure 1. [file 41598_2022_13390_MOESM1_ESM.pdf]

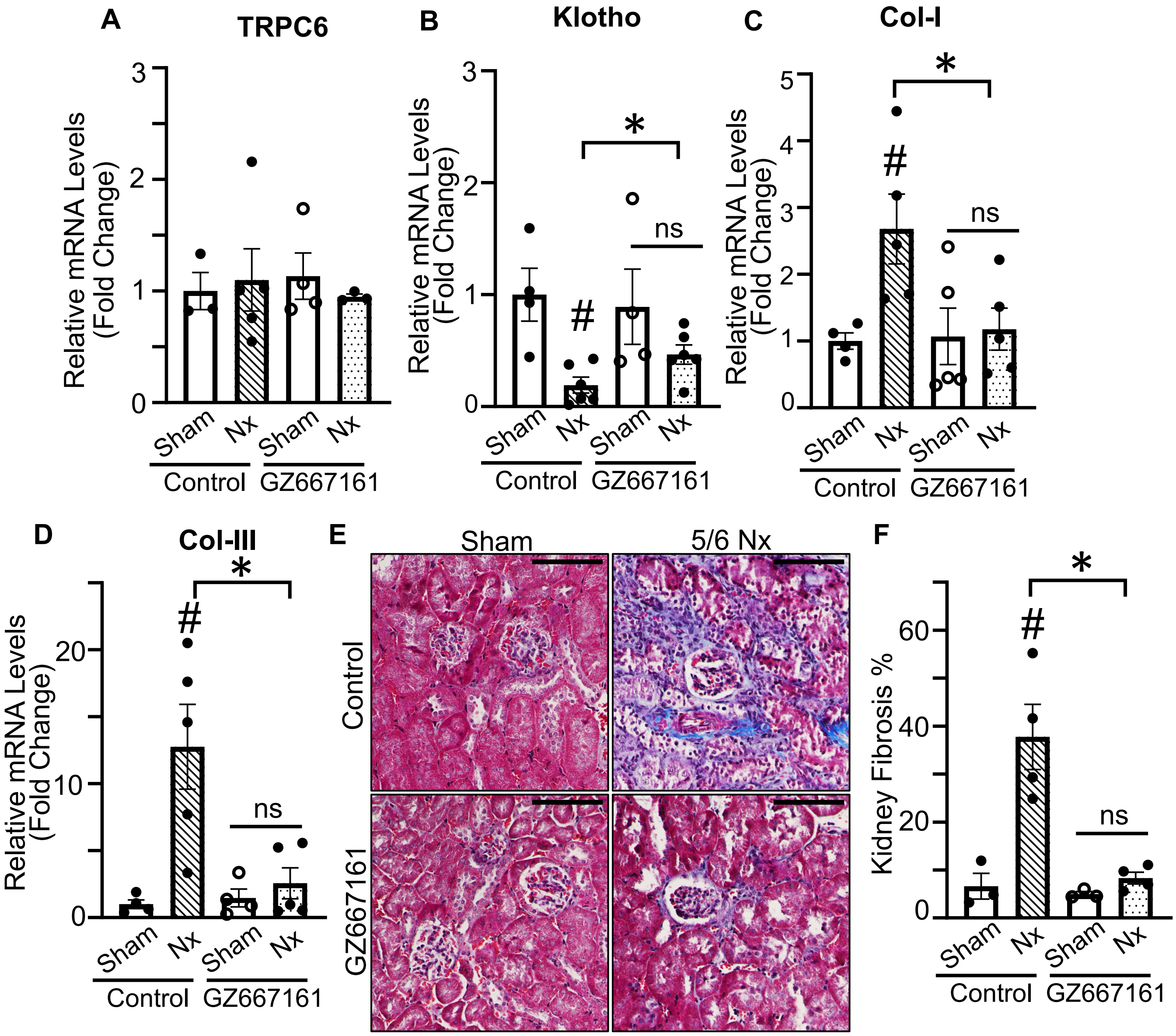

Supplementary Figure 1

Supplemental Figure 1. GZ667161 ameliorates renal fibrosis. (**A-D**) RNA from kidney tissue was isolated and RT-qPCR was performed. The following genes were examined: (**A**) TRPC6, (**B**) Klotho, (**C**) Col-I, (**D**) Col-III. (**E**) Representative trichrome staining for collagen fibers in kidneys of 5/6 Nx mice. Scale bar is 100 µm. (**F**) Quantification of kidney fibrosis percentage. Sham (n=3-5 per group); 5/6 Nx (n=4-5 per group). # $p<0.05$ vs. sham control of respective group. *$p<0.05$ between indicated groups or sham control. ns not significantly different between indicated groups. Data is expressed as mean ± SEM.
